# Early antiretroviral therapy and long-term cancer risk in HIV: 9-year outcomes from the START randomized trial

**DOI:** 10.1186/s12885-026-16188-8

**Published:** 2026-06-06

**Authors:** Ramya Ramaswami, Jacqueline A. Nordwall, Duncan Gilbert, Ellen Kitchell, Alisa Timiryasova, Ronald Mitsuyasu, Andrew N. Phillips, Jens Lundgren, Sophie Grabar

**Affiliations:** 1 HIV and AIDS Malignancy Branch, Center for Cancer Research, National Cancer Institute, National Institutes of Health, 10 Center Drive, 6N106, Bethesda, MD 20892 USA; 2 Division of Biostatistics and Health Data Science, University of Minnesota, Minneapolis, MN USA; 3 MRC Clinical Trials Unit at University College London, London, UK; 4 Division of Infectious Diseases and Geographic Medicine, University of Texas Southwestern, Dallas, TX USA; 5 CHIP Centre of Excellence for Health, Immunity, and Infections, Department of Infectious Diseases, Rigshospitalet, and Department of Clinical Medicine, Copenhagen, Denmark; 6 University of California Los Angeles Clinical AIDS Research and Education Center, Los Angeles, CA USA; 7 Institute for Global Health, University College London, London, UK; 8 Sorbonne Université, INSERM, Institut Pierre Louis d’Epidémiologie Et de Santé Publique, AP-HP, Hôpital St Antoine, Paris, 75012 France

**Keywords:** HIV-associated cancers, Infection-related cancer, ART, START, Kaposi sarcoma

## Abstract

**Background:**

Antiretroviral therapy (ART) reduces cancer risk in people with HIV (PWH) but the long-term impact of immediate ART initiation on infection-related and infection-unrelated cancers remains unclear.

**Methods:**

In the Strategic Timing of Antiretroviral Treatment (START) trial, 4684 ART-naïve adult PWH with CD4 + T-cell count above 500 cells/mm^3^ were randomized to immediate or deferred ART initiation arms (i.e., until CD4 + T-cells below 350 cells/mm^3^). In May 2015, unblinding revealed a 74% reduction in infection-related cancer risk in the immediate arm. Post-2016, participants in the deferred arm were recommended to start ART, and follow-up continued through 2021. This analysis evaluates cancer incidence (by infection-related and infection-unrelated diagnoses) between the treatment arms by time-period (overall, pre-2016 and post-2016).

**Results:**

Over a median follow-up of 9 years, 120 participants were diagnosed with cancer. The overall hazard ratio (HR) for cancer risk in the immediate versus deferred arms was 0.50 (95% CI: 0.34 to 0.73; *p* < 0.001). Pre-2016, the HR (immediate vs. deferred) was 0.33 (95% CI: 0.19 to 0.60; *P* < 0.001), while post-2016, it was 0.69 (95% CI: 0.42 to 1.13; *P* = 0.14), and *P* = 0.062 for the difference. For infection-related cancers in pre-2016 the HR was 0.24 (95% CI: 0.11 to 0.55; *P* < 0.001) and post-2016, HR was 0.53 (95% CI: 0.23 to 1.18; *P* = 0.12) and *P* = 0.18 for the difference. For infection-unrelated cancers, there was no significant difference in HR between immediate and deferred arms in either time period.

**Conclusion:**

Immediate ART initiation significantly reduces infection-related cancer risk in PWH that persists long-term.

**Clinical trial registration:**

NCT00867048.

**Supplementary Information:**

The online version contains supplementary material available at 10.1186/s12885-026-16188-8.

## Introduction

In the early 1980s, reports linked infection-related cancers, such as Kaposi sarcoma (KS) and certain non-Hodgkin lymphoma (NHL) to the human immunodeficiency virus (HIV) epidemic [1, 2]. From 1996 to the 2010 s, combination antiretroviral therapy (ART) led to a steady decline in HIV-related mortality [3–6] and the incidence of specific infection-related cancers [7–14]. During this period, uncertainty remained about the optimal timing for ART initiation in asymptomatic individuals following an initial HIV diagnosis, and whether this should be dictated by CD4 + T-cell count thresholds [15].

From 2009–2013, the Strategic Timing of Antiretroviral Treatment (START) trial randomized 4684 ART-naïve PWH with a CD4 + T-cell count > 500 cells/mm^3^ to initiate ART immediately following randomization (immediate-initiation) or defer until the CD4 + T-cell counts decline below 350 cells/mm^3^ or the onset of an AIDS-related condition (deferred-initiation) [16]. The study confirmed that those assigned to the immediate arm had a 57% reduction in the risk of the primary composite end point of serious AIDS, serious non-AIDS defining diseases (cardiovascular, renal, liver, non-AIDS cancer), or death from any cause. Specifically with regards to cancer events, the initial START study analyses from 2009 to 2015 demonstrated that immediate ART initiation reduced the risk of any cancer by 64% [16]. In May 2015, the trial was unblinded and the data safety monitoring board recommended ART initiation for the deferred group not on ART as well as extended follow-up for long-term outcomes until 2021 [17].

After a median follow-up of 3 years in the START trial, differences in types of cancer risk were noted based on the timing of ART initiation. Borges and colleagues demonstrated that immediate ART initiation significantly reduced the risk of infection-related cancer by 74%, primarily driven by decreases in cases of KS and NHL [18]. Predictors for infection-related cancers included older age and HIV viral load, but not CD4 + T-cell count. This study also evaluated infection-unrelated cancers, such as lung or prostate cancers that are less associated with immune dysregulation. Immediate ART lowered infection-unrelated cancers by 51%, though this did not reach statistical significance. Limited follow-up may have influenced these findings due to the small number of cancer events.

Here, this study examines long-term cancer rates and risk factors among participants in the START trial in the 6-year extended follow-up. We analyzed both infection-related and infection-unrelated cancers, comparing participants in the immediate and deferred treatment arms overall and in two periods: pre- and post-2016. To examine the long-term impact of ART initiation on cancer risk, we identified HIV characteristics proximal to a cancer diagnosis, evaluated the proportion of participants with multiple cancer events, and investigated causes of death among participants diagnosed with cancer.

## Methods

### Participant enrollment, ethics approval and consent

The methods and results of the START trial have been previously reported under the clinical trial NCT00867048 [16]. From 2009–2013, the Strategic Timing of Antiretroviral Treatment (START) trial randomized 4684 ART-naïve PWH with a CD4 + T-cell count > 500 cells/mm^3^ to initiate ART immediately following randomization (immediate-initiation) or defer until the CD4 + T-cell counts decline below 350 cells/mm^3^ or the onset of an AIDS-related condition (deferred-initiation) [16]. At the beginning of the extended follow-up period (January 1, 2016), 4436 randomly assigned participants (2210 in the immediate group and 2226 in the deferred group) were alive, had not withdrawn consent, and had vital status determined in the post–January 1, 2016, period (Figure S1).

The overall study oversight and ethics committee approval was provided by the University of Minnesota with additional institutional review board and ethics committee review at international the participating sites (https://www.clinicaltrials.gov/study/NCT00867048, participating sites included in Study Protocol in Supplementary Appendix). The study was conducted in accordance with the principles of the Declaration of Helsinki, the International Council for Harmonisation Good Clinical Practice guidelines. All participants provided written informed consent.

### Cancer definitions

An end-point review committee (ERC), blinded to treatment group, reviewed all reported cancer events and causes of deaths using pre-established criteria throughout the course of the study and the extended follow-up. Events considered confirmed or probable by the ERC were counted as endpoints. Deaths were classified as cancer-related if the underlying cause was designated as such per ERC review.

We categorized incident malignancies in START into infection-related and infection-unrelated cancer categories after individual review of case report forms and source documentation. This approach reflects evolving nomenclature in the field, where the three malignancies historically termed AIDS-defining cancers are increasingly classified as infection-related cancers. This terminology aligns with recent consensus recommendations to move away from the AIDS-defining cancer framework [19]. Infection-related cancers were defined as those driven by the following infectious agents: Kaposi sarcoma herpesvirus also known as human herpesvirus-8 (KSHV/HHV8, causing KS), Epstein-Barr virus (EBV causing NHL and Hodgkin Lymphoma), human papillomavirus (anal, cervical, oropharyngeal cancers and other reproductive organ cancers), hepatitis C virus (HCV) and hepatitis B virus (HBV) (liver cancer), and Helicobacter pylori (gastric cancer). All other malignancies were classified as infection-unrelated.

### Statistical analyses

Time-to-event methods, including Kaplan–Meier survival curves and Cox proportional-hazards regression models, were used to estimate the cumulative risk of cancer events for each of the randomized treatment groups. Analyses were stratified by geographic region and conducted for three time periods: (1) from randomization through 2015 (pre-2016); (2) from January 1, 2016 through 2021 (post–2016); and (3) from randomization through 2021.

Hazard ratios (HRs) comparing the immediate versus deferred groups for both pre-2016, post–2016, and overall were estimated by separate Cox models. The extent to which the HRs differed for the two time periods was assessed using a single Cox model including data from randomization through 2021, treatment group, a time-updated variable for calendar time (0 for the time from randomization through 2015, and 1 thereafter), and their interaction. To better evaluate clinical risk following ART use in the post-2016 period, participants who experienced an event pre-2016 were allowed to enter the risk set again for the second period. Post hoc analyses of the absolute risk differences (difference in event rates between the immediate versus deferred groups), 95% confidence intervals, and p-values for the difference in event rates by treatment group, by time period were calculated using Poisson regression models.

HRs for cancer types that occurred in at least 10 participants (KS, NHL, lung, and prostate) were examined. We compared HIV characteristics proximal to a cancer diagnosis by treatment arm and also evaluated the causes of death among those diagnosed with cancer during the follow up period.

Any cancer event throughout follow-up was summarized for subgroups defined according to baseline characteristics to determine which groups may benefit most from early ART. The heterogeneity of HR estimates for subgroups was assessed by including an interaction term between treatment and subgroup in expanded Cox models. For continuous subgroup variables, interactions were modeled as both categorical and continuous variables.

Statistical analyses were performed using SAS version 9.4 (SAS Institute, Inc.) or R version 3.2.3 (R Foundation for Statistical Computing). All p-values are two-sided, *p*-values < 0.05 were statistically significant. No adjustments were made for multiple comparisons due to the post-hoc and exploratory nature of these analyses.

## Results

### Baseline characteristics by arm

Between 2009 to 2021, among 4684 participants randomized to START and 41,745 person-years of follow-up (PYFU), 120 participants received an incident cancer diagnosis. The rate of both infection-related cancers and infection-unrelated cancers was 0.15/100 PYFU. At the time of randomization, participants had a median duration of HIV diagnosis of 1 year and the two study groups (immediate versus deferred arms) were well-balanced at baseline [16]. Selected baseline characteristics at time of randomization of the 120 participants with cancer and 4564 without cancer are presented in Table 1. At baseline, the median age among those subsequently diagnosed with cancer was 44 years, 19% were female and 18% were Black. The median baseline CD4 +/CD8 + T-cell ratio was lower among those with cancer as compared to those without cancer (median CD4 +/CD8 + T-cell ratio: 0.6 vs. 0.7, *P* < 0.001).

**Table 1:** Baseline characteristics by cancer event status

	**Any Cancer**	**No Cancer**	**Total**
*No. Pts*	*120*	*4564*	*4684*
Demographics			
Age (years; median, IQR)	44 (36—55)	36 (29—44)	36 (29—44)
Gender (% female)	19.2	27.0	26.8
Race (%)			
Asian	3.3	8.4	8.3
Black	18.3	30.4	30.1
Latino/Hispanic	13.3	13.6	13.6
White	63.3	44.1	44.6
Other	1.7	3.5	3.5
Geographic region			
Africa	9.2	21.6	21.3
Asia	1.7	7.8	7.6
Europe & Israel	45.8	32.5	32.9
North America	11.7	10.8	10.8
Oceana	3.3	2.3	2.3
South America	28.3	25.0	25.1
HIV History			
Likely mode of HIV infection (%)			
Sexual contact with person of same sex	57.5	55.2	55.2
Sexual contact with person of opposite sex	39.2	38.1	38.2
Injection drug use	0.8	1.4	1.4
Blood products/other/unknown	2.5	5.3	5.2
Years known to be HIV-positive (median, IQR)	1.4 (0.5—3.5)	1.0 (0.4—3.0)	1.0 (0.4—3.1)
Laboratory Results			
CD4^a^ (cells/mm^3^; median, IQR)	646 (571—760)	651 (584—765)	651 (584—765)
Nadir CD4^b^ (cells/mm^3^; median, IQR)	542 (473—647)	553 (488—654)	553 (488—654)
CD8 (cells/mm^3^; median, IQR)	1132 (893—1644)	1040 (774—1399)	1042 (777—1403)
CD4 +/CD8 + ratio (median, IQR)	0.6 (0.4—0.7)	0.7 (0.5—0.9)	0.7 (0.5—0.9)
HIV RNA (log_10_copies/mL; median, IQR)	4.3 (3.8—4.8)	4.1 (3.5—4.6)	4.1 (3.5—4.6)
Highest HIV RNA^b^ (log_10_copies/mL; median, IQR)	4.6 (3.9—5.1)	4.3 (3.7—4.8)	4.3 (3.7—4.8)
HIV RNA ≤ 400 copies/mL (%)	3.3	8.0	7.9
Medical History			
Hepatitis B (%)	2.6	2.8	2.8
Hepatitis C (%)	5.0	3.7	3.7
Smoking status (%)			
Current	38.3	31.8	32.0
Former	18.3	12.9	13.1
Never	43.3	55.2	54.9
Biomarkers			
CRP (microg/mL; median, IQR)	1.92 (0.91—4.09)	1.70 (0.73—4.00)	1.71 (0.74—4.01)
IL-6 (pg/mL; median, IQR)	1.52 (1.11—2.25)	1.39 (0.97—2.12)	1.39 (0.97—2.12)
D-dimer (µg/mL; median, IQR)	0.36 (0.28—0.56)	0.32 (0.23—0.50)	0.33 (0.23—0.50)

^a^Average of screening and baseline values

^b^Documented in participant record

Pre-2016, the percentage of follow-up time spent taking ART was 95% and 36% for the immediate and deferred groups, respectively, and the time-averaged CD4 + T-cell count difference was 199 cells/mm^3^. In the follow-up period before 2016, the median duration of ART use was 3.9 years in the immediate arm and 1.9 years in deferred arm among those with any cancer diagnosis, which were similar to those without cancer (Table S1). Post-2016, the percentage of follow-up time on ART was 97.2% and 94.1% for the immediate and deferred groups, respectively, and the CD4 + T-cell count difference was 155 cells/mm^3^, favoring the immediate arm.

### Overall cancer rates from randomization to 2021 and by time period

Among the 120 participants with cancer, 62 had infection-related and 63 had infection-unrelated cancers (Table S2). The most common cancers were KS, NHL, lung and prostate cancers. The HR (immediate/deferred) were only significant different from 1 for KS and NHL being respectively 0.12 (95% CI: 0.03 to 0.51) and 0.25 (95% CI: 0.08 to 0.75) from randomization through 2021 (Table S3).

**Fig. 1:**
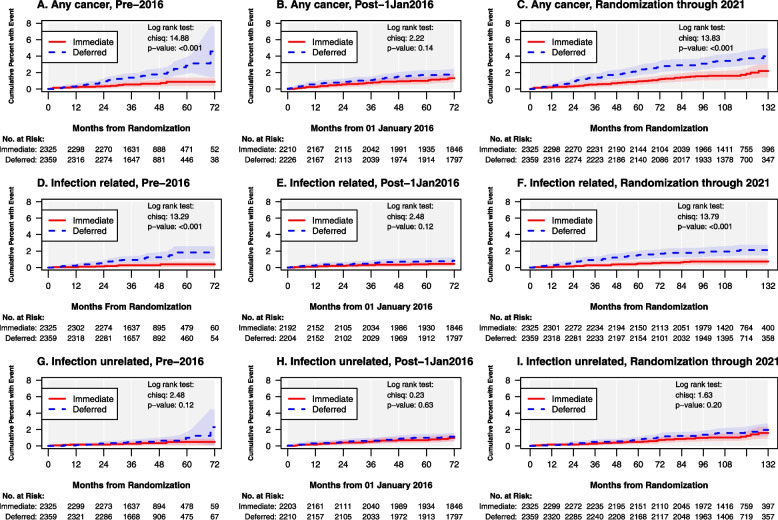
Cancer incidence rate in the immediate and treatment arms of the START study in pre-2016 period, post-2016 period and from randomization to 2021 for any cancers (**A**-**C**), infection-related cancers (**D**-**F**) and infection-unrelated cancers **G**-**I**

In the pre-2016 period, among 4684 participants, 60 were diagnosed with cancer and in the post-2016 period, in 4436 participants, 66 were diagnosed with cancer (Table 2). In all study participants with cancer from randomization to 2021, 40 were in the immediate and 80 in the deferred ART groups. The cancer rate in the deferred group was 0.51/100 PYFU in the pre-2016 period and 0.32/100 PYFU in the post-2016 period. Cancer rates in the immediate group were 0.17/100 PYFU in the pre-2016 period and 0.22/100 PYFU in the post-2016 period. Overall, from randomization to 2021, there was a lower cancer incidence in the immediate arm as compared to the deferred arm [HR 0.50 (95% CI 0.34, 0.73; *P* < 0.001)], which was also evident in the pre-2016 period (Table 2, Fig. 1 A-C). However, there was no difference in cancer incidence by immediate and deferred arms in the post-2016 period [HR 0.69 (0.42, 1.13); *P* = 0.14)]. In an analysis of the absolute difference in rates of any cancers, the difference between the two treatment groups (immediate-deferred) was −0.34 (95%CI: −0.52, −0.17) for pre-2016 time period and −0.10 (95% CI: −0.23, 0.03) for the post-2016 time period. There was a significant (*p* = 0.03) decrease in the treatment difference between the immediate and deferred groups post–January 1, 2016, compared with the pre-2016 time period (Table 3). The effect favoring the immediate group was attenuated after 2016, but there was still a large magnitude.

**Table 2:** Cancer event rates and hazard ratios by time period

	**No. Pts.**	**No.Pts. with Events(Rate per 100 py)**		**HR (95% CI)**	***p*-val**	**Ratio HR (post/pre)**	Int. *p*-val*
		**Imm.**	**Def**				
Infection-related cancer						2.19	0.18
Pre-2016	4684	7 (0.08)	29 (0.33)	0.24 [0.11, 0.55]	<0.001		
Post-Jan 1, 2016	4436	9 (0.07)	17 (0.14)	0.53 [0.23, 1.18]	0.12		
Randomization-2021	4684	16 (0.08)	46 (0.22)	0.35 [0.20, 0.61]	<0.001		
Infection-unrelated cancer						1.74	0.30
Pre-2016	4684	8 (0.09)	16 (0.18)	0.50 [0.21, 1.16]	0.11		
Post-Jan 1, 2016	4436	20 (0.16)	23 (0.19)	0.87 [0.48, 1.58]	0.64		
Randomization-2021	4684	27 (0.13)	36 (0.17)	0.75 [0.46, 1.24]	0.26		
Any cancer						2.09	0.06
Pre-2016	4684	15 (0.17)	45 (0.51)	0.33 [0.19, 0.60]	<0.001		
Post-Jan 1, 2016	4436	27 (0.22)	39 (0.32)	0.69 [0.42, 1.13]	0.14		
Randomization-2021	4684	40 (0.19)	80 (0.38)	0.50 [0.34, 0.73]	<0.001		

*Abbreviations: Imm.* Immediate, *Def.* Deferred, *HR* Hazard Ratio, *Int.* Interaction

*p-value for interaction between treatment group and time period

**Table 3:** Cancer event rates and hazard ratios by time period and arm including relative and absolute difference calculations

	**No. Pts**	**No.Pts. with Events** **(Rate per 100 py)**		**Relative Difference**			**Absolute Difference**		
		**Imm**	**Def**	**HR (95% CI)**	***p*-value**	Int. *p*-value*	**Risk Difference (95% CI)**	***p*-value**	Int. *p*-value*
Infection-related cancer						0.18			0.02
Pre-2016	4684	7 (0.08)	29 (0.33)	0.24 [0.11, 0.55]	< 0.001		-0.25 [-0.39, -0.12]	< 0.001	
Post-Jan 1, 2016	4436	9 (0.07)	17 (0.14)	0.53 [0.23, 1.18]	0.12		-0.07 [-0.15, 0.02]	0.11	
Randomization-2021	4684	16 (0.08)	46 (0.22)	0.35 [0.20, 0.61]	< 0.001		-0.14 [-0.22, -0.07]	< 0.001	
Infection-unrelated cancer						0.30			0.39
Pre-2016	4684	8 (0.09)	16 (0.18)	0.50 [0.21, 1.16]	0.11		-0.09 [-0.20, 0.02]	0.10	
Post-Jan 1, 2016	4436	20 (0.16)	23 (0.19)	0.87 [0.48, 1.58]	0.64		-0.03 [-0.13, 0.08]	0.63	
Randomization-2021	4684	27 (0.13)	36 (0.17)	0.75 [0.46, 1.24]	0.26		-0.04 [-0.12, 0.03]	0.26	
Any cancer						0.06			0.03
Pre-2016	4684	15 (0.17)	45 (0.51)	0.33 [0.19, 0.60]	< 0.001		-0.34 [-0.52, -0.17]	< 0.001	
Post-Jan 1, 2016	4436	27 (0.22)	39 (0.32)	0.69 [0.42, 1.13]	0.14		-0.10 [-0.23, 0.03]	0.13	
Randomization-2021	4684	40 (0.19)	80 (0.38)	0.50 [0.34, 0.73]	< 0.001		-0.19 [-0.30, -0.09]	< 0.001	

*Abbreviations: Imm.* Immediate, *Def.* Deferred, *HR* Hazard Ratio, *Int.* Interaction

^*^P-value for interaction between treatment group and time-period

### HIV characteristics proximal to cancer diagnoses, recurrent cancer diagnoses and second primary cancers during follow-up

The median time from HIV diagnosis to any cancer diagnosis in the immediate ART group was 6.9 years and 6.4 years in the deferred group. At the time of a cancer diagnosis, 85.0% in the immediate ART group and 51.3% in the deferred group had well-controlled HIV viral load (≤ 200 cp/mL). For any cancer diagnosis, median age prior to a cancer diagnosis was lower in the deferred group as compared to the immediate group (47 years vs. 53 years, *P* = 0.04). Furthermore, the median CD4 + T-cell and CD4 +/CD8 + ratios proximal to the cancer diagnosis was lower in deferred group as compared to the immediate ART group [median CD4 + T-cell count 590 cells/mm^3^ in deferred group vs. 800 cells/mm^3^ in the immediate ART group (*P* < 0.001), and median CD4 +/CD8 + ratio 0.5 in the deferred group vs. 1.0 in the immediate arm (*P* < 0.001), Table 4].

**Table 4:** Selected characteristics, by treatment group at the time of cancer event

**Characteristic at time of cancer**	**Infection-related Cancer**			**Infection-unrelated Cancer**			**Any Cancer**		
	**Imm ART** **Med (IQR) or %**	**Def ART** **Med (IQR) or %**	**P-val**^**1**^	**Imm ART** **Med (IQR) or %**	**Def ART** **Med (IQR) or %**	**P-val**^**2**^	**Imm ART** **Med (IQR) or %**	**Def ART** **Med (IQR) or %**	**P-val**^**3**^
No. with cancer	16	46		27	36		40	80	
Age (years)	51 (41—58)	46 (38—50)	0.13	55 (46—64)	52 (42—63)	0.37	53 (44—63)	47 (39—56)	0.04
Latest CD4 (cells/mm^3^)	606 (525—875)	579 (428—669)	0.34	881 (709—1111)	622 (512—801)	0.001	800 (579—1097)	590 (465—738)	< 0.001
Time from latest CD4 to cancer, months	1.9 (1.0—2.8)	2.0 (0.6—3.5)	0.97	2.8 (1.4—6.9)	2.6 (1.1—4.1)	0.42	2.2 (1.0—4.7)	2.3 (1.0—3.7)	0.70
Latest CD4/CD8 ratio	1.0 (0.8—1.3)	0.5 (0.3—0.7)	< 0.001	1.0 (0.8—1.5)	0.8 (0.5—1.0)	0.008	1.0 (0.8—1.3)	0.5 (0.4—0.8)	< 0.001
Time from latest CD4/CD8 to cancer, months	1.9 (1.0—4.6)	2.3 (1.1—3.5)	0.82	3.4 (1.5—11.8)	3.0 (1.4—4.2)	0.24	2.5 (1.2—8.4)	2.5 (1.3—3.7)	0.59
Latest HIV RNA (cp/mL)	40 (20—40)	2,524 (40 – 22,026)	0.002	20 (20—40)	40 (20 – 11,031)	0.02	30 (20—40)	143 (40 – 15,342)	< 0.001
Latest HIV RNA ≤ 200 cp/mL	87.5	41.3	0.001	85.2	66.7	< 0.001	85.0	51.3	< 0.001
Time from latest HIV RNA to cancer, months	1.9 (1.0—2.8)	2.0 (0.5—3.5)	0.90	2.6 (1.2—5.5)	2.6 (1.1—4.1)	0.57	1.9 (1.0—4.3)	2.3 (0.9—3.7)	0.82
Time from HIV diagnosis to cancer, years	5.6 (2.8—7.7)	5.7 (3.5—7.2)	0.77	7.8 (5.6—12.1)	7.0 (5.5—9.6)	0.09	6.9 (3.4—10.6)	6.4 (4.3—8.8)	0.52

*Abbreviations: Imm.* Immediate, *Def.* Deferred, *HR* Hazard Ratio

^1^*P*-values for difference between immediate and deferred arms for participants with infection-related cancer

^2^*P*-values for difference between immediate and deferred arms for participants with infection-unrelated cancer

^3^*P*-values for difference between immediate and deferred arms for participants with any cancer

Sixteen participants had multiple cancer events (Table 5) 8 participants had second and 5 of the 8 participants had both infection-related and infection-unrelated cancers. Among 8 participants with second primary cancers, these events occurred equally among the intervention groups and by time period. The median time from the first cancer to second primary cancer diagnosis among 8 participants was 17.5 months (range: 0–40 months). One participant in the immediate arm developed both non-Hodgkin lymphoma and oesophageal cancer concurrently 3.7 years after randomization. Cancer recurrence was seen among 6 of 62 participants (9.7%) who had infection-related cancers and 2 of 63 participants (3.2%) with infection-unrelated cancers. Among 8 participants with recurrent cancers, 6 of these individuals were in the deferred arm and all but 1 participant had onset of cancer within the pre-2016 period. The median time between recurrence among 8 participants was 11 months (range 2–36 months). Among 120 participants with cancer, 37 participants (30.8%) died during follow-up and 32 of these 37 deaths (86%) were cancer-related (Table 6), the majority were due to infection-unrelated cancers in both the immediate and deferred arms (Table S4A, S4B).

**Table 5:** Participants with second primary cancers and recurrent cancers during START follow-up

**Pt**	**Trt group**	**1st Event**			**2nd Event**			**Months from 1st event to 2nd event**
		**Type**	**Time-period**	Years to event*	**Type**	**Time-period**	Years to event*	
Second Primary Cancers								
1	Imm	Inf-related (NHL)	Post-2016	3.7	Unrelated (oesophageal)	Post-2016	3.7	0
2	Imm	Unrelated (squamous cell carcinoma)	Post-2016	6.7	Inf-related (oropharyngeal)	Post-2016	7.1	5
3	Imm	Inf-related (anal)	Pre-2016	2.5	Unrelated (colorectal)	Post-2016	5.8	40
4	Def	Unrelated (renal)	Post-2016	6.1	Inf-related (Hodgkin’s lymphoma)	Post-2016	6.2	2
5	Def	Inf-related (NHL)	Pre-2016	2.5	Unrelated (testicular)	Post-2016	5.3	34
6	Def	Inf-related (KS)	Post-2016	9.2	Inf-related (lymphoma, brain)	Post-2016	9.7	5
7	Imm	Unrelated (squamous cell carcinoma)	Post-2016	8.2	Unrelated (Basal cell carcinoma)	Post-2016	11.1	34
8	Def	Unrelated (lung)	Pre-2016	1.4	Unrelated (neuroendocrine)	Pre-2016	3.9	30
Cancer recurrence								
9	Imm	Inf-related (cervical)	Pre-2016	0.2	Inf-related (cervical)	Pre-2016	1.5	16
10	Imm	Inf-related (KS)	Pre-2016	2.0	Inf-related (KS)	Pre-2016	5.1	36
11	Def	Inf-related (NHL)	Post-2016	5.0	Inf-related (NHL)	Post-2016	5.5	6
12	Def	Inf-related (KS)	Pre-2016	2.0	Inf-related (KS)	Pre-2016	4.7	32
13	Def	Inf-related (KS)	Pre-2016	2.2	Inf-related (KS)	Pre-2016	2.4	3
14	Def	Unrelated (distal ureter)	Pre-2016	1.9	Unrelated (urethra)	Pre-2016	3.3	18
15	Def	Inf-related (NHL)	Pre-2016	1.5	Inf-related (NHL)	Pre-2016	2.0	6
16	Def	Inf-related (KS)	Pre-2016	2.0	Inf-related (KS)	Pre-2016	2.2	2

*Abbreviations: Trt* Treatment Group, *Imm.* Immediate, *Def.* Deferred, *HR* Hazard Ratio

^*^Years from randomization to event

**Table 6:** Participants who experienced a cancer event – deaths at any time point

Cause of death	Imm ART	Def ART	Overall
Infection-related: AIDS diagnosis, ongoing active disease*	1	4	5
Infection-related: other cancers*	4	3	7
COPD	0	1	1
Heart or vascular, other causes	0	1	1
Infection-unrelated malignancy**	7	13	20
Substance abuse	0	1	1
Unknown	2	0	2
Total	14	23	37

*Abbreviations: Imm*. Immediate, *Def.* Deferred

Infection-related malignancy*

Immediate ART MedDRA preferred terms: hepatocellular carcinoma (2), non-Hodgkin’s lymphoma, adenocarcinoma gastric,, oropharyngeal cancer

Deferred ART MedDRA preferred terms: non-Hodgkin’s lymphoma (3), KS, visceral, cervix, gastric, lymphoma

Infection-unrelated malignancy**

Immediate ART MedDRA preferred terms: breast, ductal adenocarcinoma of pancreas, lung adenocarcinoma, neoplasm malignant, oesophageal squamous cell carcinoma, pancreatic carcinoma, squamous cell carcinoma of lung

Deferred ART MedDRA preferred terms: lung adenocarcinoma (3), lung neoplasm malignant (2), acute myeloid leukaemia, breast, malignant melanoma, metastatic neoplasm, neoplasm malignant, oesophageal carcinoma, small cell lung cancer metastatic, transitional cell cancer of the renal pelvis and ureter

### Infection-related cancers

Throughout follow-up there were 62 infection-related cancers: 16 in immediate ART group and 46 in deferred ART group. The most common infection-related cancers were NHL (20), KS (19), Hodgkin's lymphoma (6), and anal cancer (6) (Table S2).

The rate of infection-related cancers in the deferred group was 0.33/100 PYFU during the pre-2016 period and 0.14/100 PYFU in the post-2016 period. Rates in the immediate group were 0.08 and 0.07 per 100 PYFU, respectively. The HR (immediate/deferred) was 0.24 (95% CI: 0.11 to 0.55; *P* < 0.001) pre-2016 and 0.53 (95% CI: 0.23 to 1.18; *P* = 0.12) post-2016 (*P* = 0.18 for the difference between time periods) (Table 2, Fig. 1D-F). Though there was no difference in the relative rate, the absolute difference in rates for infection-related cancers between the two treatment groups (immediate-deferred) was −0.25 (95%CI: −0.39, −0.12) for pre-2016 time period and −0.07 (95% CI: −0.15, 0.02 for the post-2016 time period. The *p*-value for the difference between these differences in rates was 0.02 (Table 3).

Among participants with infection-related cancers, though the time from HIV diagnosis to cancer onset was similar, the median CD4 +/CD8 + ratio differed between the two study arms [immediate: 1.0 (IQR: 0.8–1.3) vs. deferred: 0.5 (IQR: 0.3–0.7), *P* < 0.001, Table 4]. As expected, HIV viremia was more pronounced in the deferred group as compared with the immediate arm prior to an infection-related cancer diagnosis.

### Infection-unrelated cancers

The rate of infection-unrelated cancers in the deferred group was 0.18/100 PYFU during the pre-2016 period and 0.19/100 PYFU in the post- 2016 period. Rates in the immediate group were 0.09 and 0.16 per 100 PYFU, respectively. The HR (immediate/deferred) was 0.50 (95% CI: 0.21 to 1.16; *P* = 0.11) pre-2016 and 0.87 (95% CI: 0.48 to 1.58; *P* = 0.64) post-2016 (*p* = 0.30 for the difference between time periods) (Table 2, Fig. 1G-I).

During the entire follow up of START, there were 63 infection-unrelated cancers: 27 in the immediate ART group and 36 in the deferred ART group. The most common infection-unrelated cancers were prostate (12), lung (11), and breast (5). Similar to the trends among all cancers and for infection-related cancers, the median CD4 + T cells and CD4 +/CD8 + ratios were lower in the deferred arm as compared to the immediate arms (Table 4).

### Any cancer event by subgroups in study overall

**Fig. 2:**
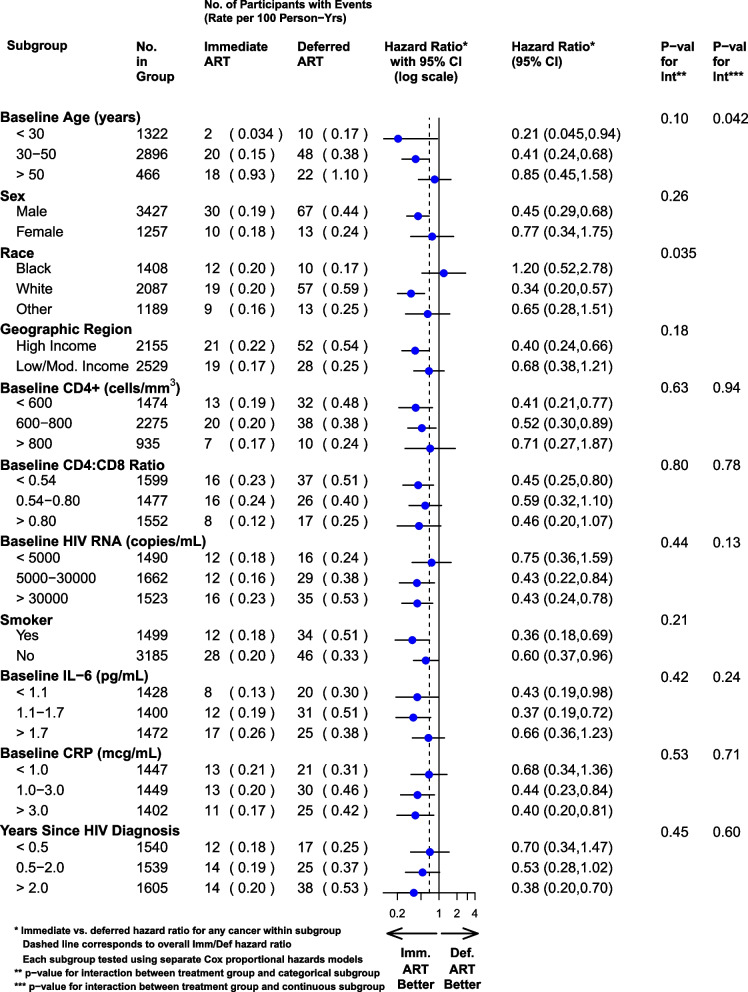
Subgroup analysis evaluating hazard ratios and events rates for immediate vs. deferred arms for any cancer during all of follow − up by baseline characteristics

Cancer events risk in the overall follow-up period by the treatment arms were evaluated by baseline demographic factors, laboratory data and inflammatory factors associated with cancer risk (Fig. 2). Across all subgroups, HRs consistently favored the immediate-initiation group with no difference across subgroups except for age and race. The HR (immediate/deferred) for cancers among those < 30 years was 0.21 compared to 0.41 for those 30–50 years and 0.85 for those > 50 years. When including age as a continuous variable, the *p*-value for interaction was 0.042. Among Black participants, the immediate vs deferred HR was 1.20 compared to 0.34 for White participants and 0.65 for other participants (*p*-value for interaction = 0.035). No other difference for heterogeneity in treatment effect by subgroups were identified.

## Discussion

In this international randomized study involving participants with early HIV infection and CD4 + T-cell count ≥ 500 cells/mm^3^, we report that immediate ART initiation reduced risk of infection-related cancers by 65% over a median follow-up of 9 years. In the post-2016 era, after participants in the deferred arm were encouraged to start ART, the differences by study arm in infection-related cancer rates still favored the immediate group but the differences narrowed and were no longer statistically significant. In contrast, the risk of infection-unrelated cancers remained unchanged overall and across either time period. Though there may not have been sufficient statistical power to detect a difference in the second time period, there are several novel key findings to emphasize with the advantage of longer follow up. First, early ART leads to a significant and sustained benefit reduction in infection-related cancer risk. Second, this benefit attenuates but persists over time following ART uptake. Third, it is important to recognize the continued burden and mortality associated with infection-unrelated cancers despite ART initiation and preserved immune function. At the time of any cancer diagnosis, uncontrolled HIV viremia was noted in close to half of the participants in the deferred arm, indicating the role of viral non-suppression in driving cancer diagnoses. CD4 + T-cell counts obtained proximal to a cancer diagnosis were consistently over 200 cells/µl. Finally, we note that a proportion of participants in both treatment groups had second primary cancers and recurrent cancers.

In the pre-2016 era, there was a lower rate of infection-related cancer rates by 76%; specifically, KS decreased by 92% and NHL by 80% in the immediate as compared to the deferred arm. Post-2016, after unblinding, there was a 47% decrease in infection-related cancer rates in the immediate arm, though this change was not significantly significant. Similarly, decreases in rates of KS and NHL were not significant between the study arms in this period. Infection-unrelated cancers decreased by 50% in the pre-2016 and 13% in the post-2016 period, but rate differences were not significant between intervention groups. The findings corroborate initial analyses conducted after 3 years of follow-up, which reported 53 cancer diagnoses [18].

The notably lower rates of KS and NHL in the immediate compared with the deferred arm during the pre-2016 period in this analysis are consistent with results from a large longitudinal observational study in North America, which demonstrated a 59% reduction in virus-related cancers for people with HIV who initiated ART earlier, with no reduction in infection-unrelated cancers [20]. Additionally, the SMART trial evaluating continuous versus intermittent ART, demonstrated lower rates of KS and NHL in the viral suppression arms [21, 22]. In our study, infection-related cancer risk was attenuated in participants in the deferred arm starting ART, but cancers still occurred in the study population over both periods. This persistence of cancer risk, with evidence of multiple cancer events in 16% of patients with cancer represents an ongoing challenge that oncologists are likely to face where PWH are diagnosed with cancer despite ART initiation, well-controlled HIV and preserved CD4 T-cell counts. Factors such as T-cell dysregulation, exposure to oncogenic viruses, smoking risk, and aging as noted in other cohort studies [23, 24], may have contributed to cancers observed over the follow-up period.

A CD4 +/CD8 + ratio less than 1 indicates immune senescence and systemic inflammation, which has been associated with HIV-associated complications [25–27], including cancers [28–30]. In a large North American observational study of 83,893 PWH including 5628 persons with cancer, the median 6-month lagged CD4 +/CD8 + ratio was 0.52 [30]. The authors reported a 10% increase in any cancer risk among those with a CD4 +/CD8 + ratio of 0.5 as compared to those with a ratio of 0.8, adjusted for covariates including age, sex, ethnicity and HIV RNA levels. In our analyses, the baseline median CD4 +/CD8 + ratio in participants with early HIV infection who subsequently developed cancer was 0.6, which was significantly lower as compared to those who did not develop cancers in the study. The median CD4 +/CD8 + ratio was 1.0 proximal to any cancer diagnosis among those in the immediate arm, whereas the median CD4 +/CD8 + ratio was significantly lower in the deferred arm. Although participants in both arms maintained high median CD4 + T cell levels from study entry to prior to a cancer diagnosis, the lower CD4 +/CD8 + ratio in the deferred arm during follow-up provides greater insights into interplay between ART initiation, T-cell dysfunction and inflammatory factors associated with cancer risk [31]. Exposure to oncogenic viruses that establish latent infection and evade host immune surveillance contribute to tumor-associated inflammation and T-cell exhaustion [32–34]. Notably PWH and KS on ART have exhibited increased frequencies of T-cells with immunosenescence phenotypes and lower naïve T-cells than PWH alone [35].

In the initial analyses after 3 years of follow-up in the START trial, Borges and colleagues highlighted older age and baseline CD8^+^ T-cell count as predictors of infection-unrelated cancer [18]. Our sub-analyses demonstrated that cancer risk reduction of immediate versus deferred ART initiation was larger for younger participants. PWH have markers of accelerated biological aging that may lead to infection-unrelated cancers that are observed in the general population [36, 37]. Immediate ART initiation may reduce cancer risk particularly in younger populations as these populations are more likely to also have infection-related cancers that are modified by ameliorating immune dysregulation with ART [38]. The subgroup analyses also highlighted that the cancer risk reduction of immediate versus deferred ART differed by race and was larger for White participants compared to Black participants. This heterogeneity by race was not noted in the long-term follow-up of the START primary endpoints [17], rather the observed disparity may be influenced by differences in access to cancer screening, or biologic factors that impact immune dysregulation and cancer susceptibility [39]. Overall, we believe that the observed interaction in these analyses should not alter the therapeutic decision of immediate initiation of ART in Black participants, but rather reinforce the need, in addition to ensuring access to ART, to study infection-unrelated cancer prevention in this population, including HIV-specific guidance for screening for malignancies [40]. Studies have also demonstrated poor uptake of cancer screening among PWH [41, 42].

There are several important limitations to consider in these analyses. First, the study evaluated the cancer risk post-hoc, and the analyses were not powered to specifically identify differences in cancer rates, such as the onset of infection-unrelated cancers, between study arms. Second, we were unable to evaluate specific cancers that occurred in less than 10 participants. Third, though there was independent review of the cancer diagnoses, specific association with infectious etiology, behavioural risk factors, cancer diagnosis due to screening procedures, or genetic predisposition to cancer could not be fully ascertained in all cases. Fourth, the baseline median age among participants was 36 years and the length of follow-up may have been insufficient to capture cancers that may occur in participants over 60 years of age. Lastly, there were no adjustment for multiple comparisons within these analyses. Despite these limitations, the robust randomized study design permits important conclusions to reduce cancer risk in PWH with high CD4 + T-cell counts during early HIV infection.

## Conclusion

In conclusion, immediate initiation of ART significantly reduces the risk of infection-related cancers in PWH over long-term follow-up, supporting current guidelines recommending early treatment initiation. However, infection-unrelated cancer risk remains unchanged, underscoring the need for comprehensive cancer prevention strategies that address the multifactorial nature of cancer risk in PWH. As PWH continue to live longer with effective ART, efforts to mitigate cancer risk will become increasingly important in optimizing long-term health outcomes.

## Supplementary Information

Supplementary Material 1.

## Data Availability

Access to data can be received by submitting a proposal via the INSIGHT website (www.insight-trials.org) that will be reviewed by the networks scientific steering committee. The scientific secretariat can be contacted directly using this mail address: [insightsscsec.rigshospitalet@regionh.dk](mailto:insightsscsec.rigshospitalet@regionh.dk).
