# Supplementary material for: Early antiretroviral therapy and long-term cancer risk in HIV: 9-year outcomes from the START randomized trial
Source: BMC Cancer. 2026 Jun 6;26:963. doi: 10.1186/s12885-026-16188-8 (PMC13459324; doi:10.1186/s12885-026-16188-8)
Supplement: Supplementary file 1 [file 12885_2026_16188_MOESM1_ESM.docx]

**Supplementary Figure 1. Flow diagram of the START study cohort from randomization to 01Jan2016 period**

**Supplementary Table 1. Selected characteristics by treatment group at the end of 2015 (before 01Jan2016) for those with follow-up in the post-01Jan2016 period.**

|  | **Any Cancer** | | **No Cancer** | |
| --- | --- | --- | --- | --- |
| **Characteristic** | **Immediate ART**  **Median [IQR] or No. (%)** | **Deferred ART**  **Median [IQR] or No. (%)** | **Immediate ART**  **Median [IQR] or No. (%)** | **Deferred ART**  **Median [IQR] or No. (%)** |
| **No. alive and not withdrawn** | **35** | **73** | **2175** | **2153** |
| Follow-up accrued, years | 3.9 [2.9, 5.1] | 4.4 [3.5, 5.4] | 3.6 [2.9, 4.8] | 3.6 [2.9, 4.7] |
| Latest CD4 (cells/mm^3^) | 751 [579, 941] | 592 [458, 732] | 872 [707, 1082] | 646 [511, 827] |
| Latest CD4:CD8 ratio | 0.94 [0.73, 1.34] | 0.70 [0.42, 0.97] | 1.19 [0.90, 1.56] | 0.75 [0.52, 1.06] |
| Latest HIV RNA ≤ 200 cp/mL | 32 (91.4%) | 61 (83.6%) | 2030 (93.4%) | 1557 (72.3%) |
| Initiated ART | 34 (97.1%) | 68 (93.2%) | 2152 (98.9%) | 1819 (84.5%) |
| Latest CD4 before ART   initiation, (cells/mm^3^) | 634 [575, 757] | 424 [324, 555] | 648 [580, 764] | 460 [345, 602] |
| Duration of ART use, years | 3.9 [2.8, 4.8] | 1.9 [0.5, 3.0] | 3.5 [2.7, 4.6] | 0.6 [0.3, 2.3] |
| % of Follow-up time on ART | 93.1% | 44.1% | 94.5% | 35.2% |

**Supplementary Table 2. Number of participants with a cancer event by time period and treatment assignment**

|  | **Pre-2016** | | | **Post 1Jan2016** | | | **Total** | | |
| --- | --- | --- | --- | --- | --- | --- | --- | --- | --- |
| **Event** | **Imm** | **Def.** | **Total** | **Imm** | **Def.** | **Total** | **Imm** | **Def.** | **Total** |
| **Any Infection-related Cancer** | **7** | **29** | **36** | **9** | **17** | **26** | **16** | **46** | **62** |
| Anal | 1 | 2 | 3 | 0 | 3 | 3 | 1 | 5 | 6 |
| Cervical carcinoma | 1 | 0 | 1 | 0 | 1 | 1 | 1 | 1 | 2 |
| Gastric | 0 | 1 | 1 | 1 | 0 | 1 | 1 | 1 | 2 |
| Liver | 0 | 0 | 0 | 2 | 0 | 2 | 2 | 0 | 2 |
| Kaposi's sarcoma | 1 | 13 | 14 | 1 | 4 | 5 | 2 | 17 | 19 |
| Lymphoma, brain | 0 | 1 | 1 | 0 | 1 | 1 | 0 | 2 | 2 |
| Lymphoma, Hodgkin's | 2 | 1 | 3 | 2 | 1 | 3 | 4 | 2 | 6 |
| Lymphoma, non-Hodgkin's | 2 | 10 | 12 | 2 | 6 | 8 | 4 | 16 | 20 |
| Oropharyngeal | 0 | 0 | 0 | 1 | 0 | 1 | 1 | 0 | 1 |
| Uterine | 0 | 1 | 1 | 0 | 0 | 0 | 0 | 1 | 1 |
| Death, lymphoma (type unknown) | 0 | 0 | 0 | 0 | 1 | 1 | 0 | 1 | 1 |
|  |  |  |  |  |  |  |  |  |  |
| **Any Infection-unrelated Cancer** | **8** | **16** | **24** | **20** | **23** | **43** | **27** | **36** | **63** |
| Basal cell carcinoma | 0 | 0 | 0 | 0 | 1 | 1 | 0 | 1 | 1 |
| Bladder | 1 | 0 | 1 | 0 | 0 | 0 | 1 | 0 | 1 |
| Breast | 1 | 1 | 2 | 2 | 1 | 3 | 3 | 2 | 5 |
| Colorectal | 0 | 0 | 0 | 1 | 2 | 3 | 1 | 2 | 3 |
| Endometrial | 0 | 0 | 0 | 1 | 0 | 1 | 1 | 0 | 1 |
| Fibrosarcomas | 1 | 0 | 1 | 0 | 0 | 0 | 1 | 0 | 1 |
| Extragonadal primary germ cell | 0 | 0 | 0 | 0 | 1 | 1 | 0 | 1 | 1 |
| Intestinal adenocarcinoma | 0 | 0 | 0 | 1 | 0 | 1 | 1 | 0 | 1 |
| Leiomyosarcomas | 0 | 1 | 1 | 0 | 0 | 0 | 0 | 1 | 1 |
| Leukaemias | 0 | 2 | 2 | 0 | 1 | 1 | 0 | 3 | 3 |
| Lung | 2 | 2 | 4 | 1 | 6 | 7 | 3 | 8 | 11 |
| Multiple myeloma | 1 | 0 | 1 | 0 | 0 | 0 | 1 | 0 | 1 |
| Oesophageal malignant | 0 | 0 | 0 | 1 | 1 | 2 | 1 | 1 | 2 |
| Pancreatic | 0 | 0 | 0 | 2 | 0 | 2 | 2 | 0 | 2 |
| Prostate | 2 | 3 | 5 | 3 | 4 | 7 | 5 | 7 | 12 |
| Renal | 0 | 0 | 0 | 2 | 1 | 3 | 2 | 1 | 3 |
| Renal, pelvis, ureter | 0 | 1 | 1 | 0 | 0 | 0 | 0 | 1 | 1 |
| Skin melanoma | 0 | 1 | 1 | 2 | 0 | 2 | 2 | 1 | 3 |
| Secondary liver | 0 | 1 | 1 | 0 | 0 | 0 | 0 | 1 | 1 |
| Squamous cell carcinoma | 0 | 1 | 1 | 2 | 0 | 2 | 2 | 1 | 3 |
| Testicular | 0 | 2 | 2 | 0 | 1 | 1 | 0 | 3 | 3 |
| Thyroid | 0 | 1 | 1 | 0 | 0 | 0 | 0 | 1 | 1 |
|  |  |  |  |  |  |  |  |  |  |
| Death |  |  |  |  |  |  |  |  |  |
| *Death, Breast* | 0 | 0 | 0 | 1 | 0 | 1 | 1 | 0 | 1 |
| *Death, Secondary liver* | 0 | 0 | 0 | 0 | 1 | 1 | 0 | 1 | 1 |
| *Death, Lung* | 0 | 0 | 0 | 0 | 1 | 1 | 0 | 1 | 1 |
| *Death, Myelomas* | 0 | 0 | 0 | 0 | 1 | 1 | 0 | 1 | 1 |
| *Death, Urinary* | 0 | 0 | 0 | 0 | 1 | 1 | 0 | 1 | 1 |
| *Death, Unknown origin* | 0 | 0 | 0 | 1 | 0 | 1 | 1 | 0 | 1 |
|  |  |  |  |  |  |  |  |  |  |
| **Any Cancer** | **15** | **45** | **60** | **27** | **39** | **66** | **40** | **80** | **120** |
|  | | | | | | | | | |
| Summary is for the first infection-related event and first infection-unrelated event in each time period. Some participants experienced both types of events in a given period or an event in both time periods. | | | | | | | | | |

| **Supplementary Table 3. Event Rates and Hazard Ratios, Pre-2016 and Post-January 1, 2016** | | | | | |
| --- | --- | --- | --- | --- | --- |
|  | **No.Pts. with Events (Rate per 100 py)** | |  |  |  |
|  | **Imm.** | **Def** | **HR (95% CI)** | **p-val** | **Int. p-val*** |
| **Kaposi's sarcoma** |  |  |  |  | 0.45 |
| Pre-2016 | 1 (0.01) | 13 (0.15) | 0.08 [0.01, 0.59] | 0.013 |  |
| Post-Jan 1, 2016 | 1 (0.01) | 4 (0.03) | 0.25 [0.03, 2.20] | 0.21 |  |
| Randomization-2021 | 2 (0.01) | 17 (0.08) | 0.12 [0.03, 0.51] | 0.004 |  |
|  |  |  |  |  |  |
| **Lymphoma, non-Hodgkin's** |  |  |  |  | 0.65 |
| Pre-2016 | 2 (0.02) | 10 (0.11) | 0.20 [0.04, 0.92] | 0.039 |  |
| Post-Jan 1, 2016 | 2 (0.02) | 6 (0.05) | 0.33 [0.07, 1.66] | 0.18 |  |
| Randomization-2021 | 4 (0.02) | 16 (0.08) | 0.25 [0.08, 0.75] | 0.013 |  |
|  |  |  |  |  |  |
| **Lung** |  |  |  |  | 0.22 |
| Pre-2016 | 2 (0.02) | 2 (0.02) | 1.00 [0.14, 7.14] | 1.00 |  |
| Post-Jan 1, 2016 | 1 (0.01) | 6 (0.05) | 0.16 [0.02, 1.35] | 0.09 |  |
| Randomization-2021 | 3 (0.01) | 8 (0.04) | 0.37 [0.10, 1.40] | 0.14 |  |
|  |  |  |  |  |  |
| **Prostate** |  |  |  |  | 0.92 |
| Pre-2016 | 2 (0.03) | 3 (0.05) | 0.67 [0.11, 3.99] | 0.66 |  |
| Post-Jan 1, 2016 | 3 (0.03) | 4 (0.05) | 0.75 [0.17, 3.35] | 0.71 |  |
| Randomization-2021 | 5 (0.03) | 7 (0.04) | 0.72 [0.23, 2.26] | 0.57 |  |
|  |  |  |  |  |  |
|  | | | | | |

* p-value for interaction between treatment group and time-period

**Supplementary Table 4:** **Causes of death for START participants**

**Table 4A: Causes of death for START participants who experienced a cancer event – deaths through 1^st^ Jan 2016**

| **Cause of death** | **Imm ART** | **Def ART** | **Overall** |
| --- | --- | --- | --- |
| Infection-related: AIDS diagnosis, ongoing active disease* | 1 | 3 | 4 |
| Infection-related: other cancers* | 0 | 1 | 1 |
| Heart or vascular, other causes | 0 | 1 | 1 |
| Infection-unrelated malignancy** | 2 | 2 | 4 |
| Unknown | 1 | 0 | 1 |
| Total | 4 | 7 | 11 |

**Infection-related malignancy***

Immediate ART: non-Hodgkin’s lymphoma

Deferred ART: non-Hodgkin’s lymphoma (2), KS visceral, gastric

**Infection-unrelated malignancy****

Immediate ART MedDRA preferred terms: lung adenocarcinoma, squamous cell carcinoma of lung

Deferred ART MedDRA preferred terms: malignant melanoma, small cell lung cancer metastatic

**Table 4B: Causes of death for START participants who experienced a cancer event – deaths after 1^st^ Jan 2016**

| **Cause of death** | **Imm ART** | **Def ART** | **Overall** |
| --- | --- | --- | --- |
| Infection-related: AIDS diagnosis, ongoing active disease* | 0 | 1 | 1 |
| Infection-related: other cancers* | 4 | 2 | 6 |
| COPD | 0 | 1 | 1 |
| Infection-unrelated malignancy** | 5 | 11 | 16 |
| Substance abuse | 0 | 1 | 1 |
| Unknown | 1 | 0 | 1 |
| Total | 10 | 16 | 26 |

**Infection-related malignancy***

Immediate ART: hepatocellular carcinoma (2), adenocarcinoma gastric, , oropharyngeal cancer

Deferred ART: non-Hodgkin’s lymphoma, cervix, lymphoma,

**Infection-unrelated malignancy****

Immediate ART death MedDRA preferred terms: breast, ductal adenocarcinoma of pancreas, neoplasm malignant, oesophageal squamous cell carcinoma, pancreatic carcinoma

Deferred ART MedDRA preferred terms: lung adenocarcinoma (3), lung neoplasm malignant (2), acute myeloid leukaemia, breast, metastatic neoplasm, neoplasm malignant, oesophageal carcinoma, transitional cell cancer of the renal pelvis and ureter
